# The Role of Sclerostin in Rheumatic Diseases: A Review

**DOI:** 10.3390/jcm12196248

**Published:** 2023-09-28

**Authors:** Łukasz Jaśkiewicz, Grzegorz Chmielewski, Jakub Kuna, Tomasz Stompór, Magdalena Krajewska-Włodarczyk

**Affiliations:** 1 Department of Human Physiology and Pathophysiology, School of Medicine, Collegium Medicum, University of Warmia and Mazury in Olsztyn, 10-082 Olsztyn, Poland; 2 Department of Rheumatology, School of Medicine, Collegium Medicum, University of Warmia and Mazury in Olsztyn, 10-900 Olsztyn, Poland; 3 Department of Nephrology, Hypertension and Internal Medicine, University of Warmia and Mazury in Olsztyn, 10-516 Olsztyn, Poland

**Keywords:** rheumatoid arthritis, systemic lupus erythematosus, ankylosing spondylitis, psoriatic arthritis, sclerostin, Wnt/β-catenin pathway

## Abstract

Systemic connective tissue disorders constitute a heterogenous group of autoimmune diseases with the potential to affect a range of organs. Rheumatoid arthritis (RA) is a chronic, progressive, autoimmune inflammatory disease affecting the joints. Systemic lupus erythematosus (SLE) may manifest with multiple system involvement as a result of inflammatory response to autoantibodies. Spondyloarthropathies (SpAs) such as ankylosing spondylitis (AS) or psoriatic arthritis (PsA) are diseases characterised by the inflammation of spinal joints, paraspinal tissues, peripheral joints and enthesitis as well as inflammatory changes in many other systems and organs. Physiologically, sclerostin helps to maintain balance in bone tissue metabolism through the Wnt/β-catenin pathway, which represents a major intracellular signalling pathway. This review article aims to present the current knowledge on the role of sclerostin in the Wnt/β-catenin pathway and its correlation with clinical data from RA, SLE, AS and PsA patients.

## 1. Introduction

Systemic connective tissue disorders constitute a heterogenous group of autoimmune diseases with the potential to affect a range of organs. The presence of autoantibodies can be recognised as their characteristic feature. They are usually disease-specific, and so they have been included into the classification criteria [1,2].

Rheumatoid arthritis (RA) is a chronic autoimmune disease involving the joints, but may also be associated with serious systemic symptoms, e.g., interstitial lung disease or hematologic disorders [3,4,5]. It is a progressive disease and the persistent inflammatory process leads to cartilage damage and the formation of erosions, gradually causing disability [6].

Systemic lupus erythematosus (SLE) is a systemic autoimmune disorder, which may involve multiple systems. Its exact aetiology is still unclear [7]. The disease may have different clinical presentations, which complicates prognostic assessment in this patient group [8,9]. It has been demonstrated that environmental and genetic factors, through their mutual interaction, may be implicated in triggering the immune response, resulting in excessive autoantibody production, which leads to inflammation-mediated tissue and organ injury. SLE is characterised by the presence of antibodies targeted at nuclear and cytoplasmic antigens [10,11].

Spondyloarthropathies (SpAs) are inflammatory diseases involving spinal joints, peripheral joints and tendons. These include the following: ankylosing spondylitis (AS), psoriatic arthritis (PsA), reactive arthritis and non-specific inflammatory bowel disease-associated arthritis [12,13,14,15].

Sclerostin, a glycoprotein produced and released primarily by mature osteocytes, is an inhibitor of Wnt/β-catenin pathway-dependent osteoblast proliferation and differentiation from mesenchymal stem cells [16]. Physiologically, sclerostin is a regulator of bone tissue metabolism. As a result of mechanical loading and microtraumas, and with oestrogen deficiency, the release of sclerostin is inhibited, which stimulates the processes of bone formation and repair [17,18,19,20]. With age, there is an increase in plasma sclerostin concentration in both sexes, and this may be associated with age-related osteoporosis [21]. Apart from inhibiting the Wnt/β-catenin pathway, sclerostin can stimulate RANKL secretion in osteocytes, osteoclastogenesis and bone resorption [22,23]. Sclerostin was originally discovered as a result of studies on inactivating mutations in the coding and enhancer regions of the *SOST* gene [24]. The function of sclerostin as an inhibitor of osteogenesis has been confirmed in a study involving transgenic mice. Sclerostin knock-out (SOST KO) mice showed high bone mass and increased bone formation and bone strength [25,26], whereas animals with sclerostin overexpression presented with low bone mass and bone fragility [27].

To date, only a few publications have investigated the role of sclerostin as a potential biomarker in rheumatic diseases.

## 2. Wnt/β-Catenin Pathway

**Figure 1:**
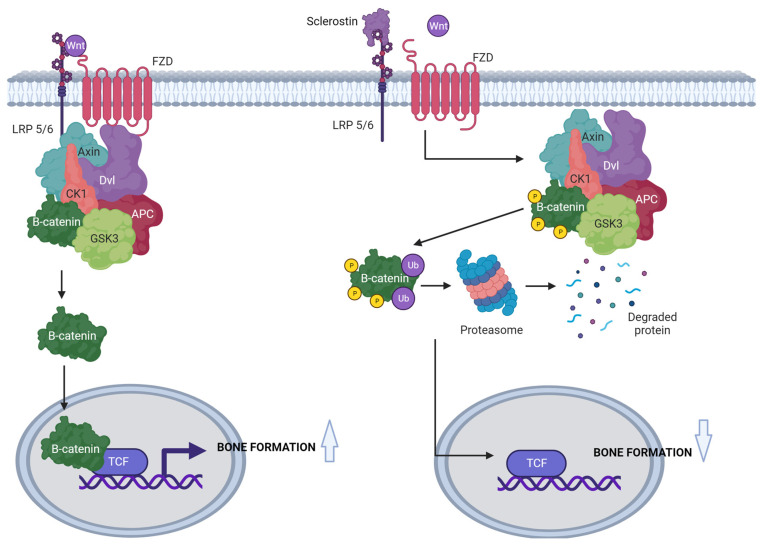
The effect of sclerostin on the Wnt/β-catenin pathway. When the Wnt signalling pathway is not inhibited by sclerostin, it leads to increased bone formation. Sclerostin inhibits the canonical Wnt-signalling pathway through its binding to the Wnt LRP 5/6 co-receptors and leads to decreased bone formation. APC: adenomatous polyposis coli; CK1α: casein kinase 1 α; Dvl: Dishevelled protein; FZD: Frizzled receptor; GSK3: glycogen synthase kinase 3; LRP 5/6: low-density lipoprotein receptor-related protein 5/6; P: phosphorus; Ub: ubiquitin; TCF: transcription factor. Created using BioRender.com (accessed on 16 August 2023).

The Wnt pathway is recognised as a major intracellular signalling pathway that is also in osteocytes [28,29]. It may involve the activation of the best-studied, β-catenin-dependent, canonical pathway, or a few non-canonical pathways [30]. The canonical pathway regulates the activity of T-cell factor (TCF), impacting the embryogenesis, differentiation and proliferation of cells [31]. Wnt proteins are implicated in initiating intracellular signalling pathways by binding to specific Frizzled transmembrane receptors, showing a high degree of affinity to Wnt proteins [32]. Apart from the interaction of a Wnt protein with a Frizzled receptor, the activation of the signalling pathway requires the binding of a co-receptor from the family of low-density lipoprotein receptor (LDLR)-related proteins, particularly LRP5 and LRP6. This leads to the formation of a trimeric complex capable of signal transduction [33]. Additionally, Dishevelled (Dvl) protein [34] and axin [35] bind to the cytoplasmic parts of the Frizzled receptor and LRP co-receptor, respectively. The activation of the complex is associated with the heterodimerisation of Dvl proteins and axin, which results in the re-configuration of the complex and the activation and detachment of β-catenin [36]. Free, active β-catenin accumulates in the cytoplasm and is then transported to the cell nucleus, where it binds to the TCF protein, constituting one of the major transcription factors. The interaction of β-catenin with CF leads to chromatin remodelling, adjacent to the TCF binding site, and is a key stimulator in commencing gene transcription [37]. With no signal stimulating the Wnt pathway, the amount and activity of β-catenin are limited by the operation of the so-called destruction complex, formed by axin (here, unbound to the LRP co-receptor), protein APC (adenomatous polyposis coli) and two serine-threonine kinases: CKIα (casein kinase 1α) and GSK3 (glycogen synthase kinase 3). The activity of kinases in the axin/APC/CKIα/GSK3 complex leads to β-catenin phosphorylation, its identification via β-TrCP ligase (β-transducing repeat-containing protein ligase) and ubiquitination, with its ultimate degradation in proteasomes [38]. The inhibition of the Wnt signalling pathway may involve extracellular inhibitors of Wnt activators such as sFRP (secreted Frizzled-related protein) [39] and WIF (Wnt inhibitory factor) [40], as well as inhibitors of the LRP co-receptor including Wise proteins [41], Dkk-1 (Dickkopf-related protein-1) [42] and sclerostin [43]. See Figure 1.

## 3. The Role of Sclerostin in Rheumatoid Arthritis

Rheumatoid arthritis (RA) involves, as its integral component, disorders of bone tissue metabolism manifested by erosions, periarticular osteoporosis and generalised osteoporosis [4,44,45]. The stage of the disease and the rate of bone involvement progression depend on the intensity of bone resorption and inadequate bone formation, which may be conditioned by a range of factors connected with inflammatory joint disease, such as pro-inflammatory cytokine overproduction, limited physical activity or medications used. They may also include well-recognised risk factors for osteoporosis, i.e., old age, endocrine disorders, genetic susceptibility, low peak bone mass, nutrient-deficient diet and smoking [46,47]. The ethology of RA is still not entirely clear. Many factors, such as genetic background, smoking or infections, play a role in the process of converting arginine into citrulline by the Peptidyl Arginine Deiminase 4 (PADI4) enzyme [48,49]. A genetic predisposition was identified, which was located in a common epitope in the HLA-DRB1 locus of major histocompatibility complex (MHC) class II antigens [50]. The key factor in the development of RA is the occurrence of anti-citrullinated protein antibody (ACPA) and/or rheumatoid factor (RF) produced by plasma cells of the synovial membrane [51], which results in the stimulation of monocytes, mast cells and dendritic cells, but also Th1, Th17, B lymphocytes and plasma cells, to produce mediators of the inflammatory reaction [52]. RF interacts directly with the Fc region of IgG and forms immune complexes that increase vascular permeability and have a chemotactic effect [53]. ACPA is directed against, among others, the following: citrullinated filaggrin, fibrinogen, vimentin and α-enolase, which activates the complement system and induces the secretion of TNF-α by macrophages [54]. T lymphocytes recognise the antigen through antigen-presenting cells (APCs) and trigger a specific response. The pathogenesis of RA involves Th1 lymphocytes producing IFN-γ and Th17 lymphocytes, which are often found in the synovium of RA patients and rarely in the synovium of the joints of healthy people. Th17 lymphocytes, through the secretion of IL-17 and IL-22, strongly stimulate macrophages to secrete pro-inflammatory cytokines such as TNF-α, IL-1 and IL-6 [55,56,57]. B lymphocytes differentiate into antibody-producing plasma cells, and also produce pro-inflammatory cytokines and play the role of antigen-presenting cells and cells regulating the humoral response [58]. Fibroblast-like synoviocytes (FSCs) are activated and, together with other inflammatory cells, produce RANKL [59]. Ultimately, this leads to increased production of osteoclasts and the formation of erosions. In RA, in particular, macrophages produce large amounts of pro-inflammatory cytokines, i.e., TNF-alpha, IL-6, IL-1, IL-15, IL-18, IL-32 and chemotactic factors [57]. TNF-α increases the expression of sclerostin in primary osteocytes and also enhances the formation of osteoclasts induced by RANKL, which promotes the formation of erosions [60,61].

The risk for cardiovascular diseases observed in RA patients is significantly higher than in the general population [5,6] and exceeds the risk assessed according to traditional factors contributing to arteriosclerosis [7,8,9]. Chronic inflammation in RA is associated with endothelial dysfunction and the formation of arteriosclerotic lesions. Advanced arteriosclerosis is marked by particularly severe arterial calcification [10,11,12], mainly involving the intimal layer [13]; calcification of the medial layer (Mönckeberg’s calcification) may develop independently of changes observed in arteriosclerosis [14]. While intimal calcifications occupy focal areas and are mainly found in arteriosclerotic plaque, calcifications in the medial layer display a linear pattern [15].

In the few studies conducted to date investigating the association between sclerostin and the course of RA, serum sclerostin concentrations were higher [62,63,64], the same [65,66] or lower [67] when compared to the results obtained in control groups of healthy individuals. The discrepancies may have been due to differences between patient groups participating in the study. They could have also been due to the effect of using different assays to determine sclerostin concentrations. Somewhat unexpectedly, the sclerostin concentration was observed to rise during successful therapy with etanercept [68] and tocilizumab [69], while it decreased with greater disease activity, as measured via DAS28 [62,63,64,68], the number of tender joints [65] and an increase in the C-reactive protein (CRP) level [64,66]. There seems to be no association between sclerostin concentration and advanced stage of the disease radiologically [63,65,70], although there was a significant correlation reported between its level and local occurrence of severe osteoarticular lesions, as assessed using the Larsen scoring system [64]. Moreover, there was no correlation found between sclerostin concentration and bone mineral density in the forearm or femoral neck bone in RA patients [64,71]. In a study by Paccou et al., there was an association identified between increased BMD of the lumbar region and rise in sclerostin concentration, similarly to the control group, where a high sclerostin level was consistent with high BMD values in the femoral neck bone [71]. These results may suggest the involvement of total mass of active osteocytes and potential engagement of other excited cells, including synoviocytes, in the process of sclerostin production and release [72]. 

In a study by Wehmeyer et al. in an arthritis model in hTNFtg mice, SOST expression was not only present in osteocytes but was TNF-α-induced in FLS cells, which were the main source of sclerostin. Interestingly, also in this study, sclerostin seemed to exert a protective effect, as the administration of sclerostin inhibitor led to greater joint swelling, weaker grip and progression of bone lesions in the joints. In a mouse model of antigen (G6PI)-induced, partially TNF-α-dependent arthritis, sclerostin was found to have no effect on bone loss; however, it diminished the disease activity in a TNF-α-independent, serum-derived model in K/BxN mice. Additionally, the administration of recombinant sclerostin inhibited, through LRP6, the activity of TNF-α-induced (but not IL-1-induced) MAP: p38 and ERK kinases, and as a result, the activity of NFκB in synoviocytes from mice and in those obtained from RA patients as well. Moreover, it was associated with decreased expression of the RANK ligand on human FLS [72]. In a study using a collagen-induced arthritis model, using sclerostin inhibitor prevented the loss of total bone mass, having no effect on the formation of local bone erosions [73]. In another study, although blocking sclerostin in hTNFtg mice inhibited bone destruction, this was only after the additional administration of TNF-α inhibitor [74]. Of note, animal models of arthritis used in the studies do not truly represent human rheumatoid arthritis, and so the reported discrepancies in the results may be due to the dominant activity of particular cytokines in these models.

## 4. The Role of Sclerostin in Systemic Lupus Erythematosus

Systemic lupus erythematosus (SLE) may cause disorders of the locomotor system which usually include painful conditions of joints and muscles, tendonitis or tenosynovitis. However, periarticular structures usually remain unaffected [75]. Tendonitis, tenosynovitis or capsulitis may lead to Jaccoud arthropathy, which is characterised by joint deformity, but, unlike in RA, no erosions are observed [76]. In the pathophysiology of SLE, we observed increased formation of autoantigen–antibody complexes by autoreactive B lymphocytes, which were then recognised by plasmacytoid dendritic cells. This leads to the stimulation of Toll-like receptor [TLR]-7 and TLR-9-dependent pathways, as well as the secretion of endogenous IFN-α [77]. This causes the additional stimulation of both groups of lymphocytes: B and T. Hyperactivity of T lymphocytes, in particular the CD4+ subpopulation [78], may affect the Wnt/β-catenin pathway, which has also been described in animal models of lupus nephritis [79]. 

In the few studies conducted to date, aiming to assess the association between sclerostin and the course of SLE, the serum sclerostin concentration was found to be elevated. Fayed et al. compared a group of 100 patients with a control group of 50 and demonstrated increased sclerostin concentration in patients with SLE and its statistically significant correlation with proteinuria in these patients. This confirms a key role of Wnt/β-catenin signalling in SLE pathogenesis, which means that sclerostin may become a potential biomarker of lupus nephritis in the future [80]. The research to date has shown that the Wnt/β-catenin signalling pathway plays a role in cell protection against stress factors and apoptosis [81]. However, excessive and long-lasting activation of Wnt/β-catenin signalling may enhance the expression of matrix metalloproteinases (MMPs), particularly MMP-2 and MMP-9, in glomeruli. Metalloproteinases, through the degradation of the matrix, lead to the loss of membrane and extracellular matrix integrity, and, moreover, may lead to renal fibrosis and progressive renal dysfunction [82]. Garcia-de los Ríos et al. reported on the association of sclerostin with the presence of atherosclerotic plaque in the carotid arteries of women with SLE [83]. To date, there have been reports on a correlation between sclerostin concentration and arterial calcification in RA [71].

## 5. The Role of Sclerostin in Psoriatic Arthritis

Psoriatic arthritis (PsA) is a chronic disease manifesting with synovitis, enthesitis and dermatitis, with no rheumatoid factor present. The disease has a heterogenous clinical presentation regarding the number of joints affected and the degree of their damage, as well as the severity of skin lesions, if seen, as these may be absent [84,85]. IL-17 plays an important role in the pathogenesis of PsA, affecting bone formation in inflammatory places as a result of mechanical injury, as is the case with entheses in animal models of SpA [86]. In addition, TNF-α, IL22 and IL23 play an important role. IL23, which is secreted mainly by macrophages and dendritic cells, but also by keratinocytes, is primarily responsible for the induction of Th17 cells [87]. Th17 cells produce IL-17, which enhances the production of pro-inflammatory cytokines such as IL1-β, IL-6 and TNF-alpha by synovial fibroblasts and macrophages [88]. Ultimately, this leads to the destruction of bone tissue as a result of the inflammatory process. Moreover, IL-23 promotes the expression of RANK, which leads to the differentiation of precursor cells toward osteoclasts, which intensifies bone tissue damage [89]. In turn, IL-22 has a bone-forming effect by inducing the formation of osteoblasts [90]. 

Fassio et al. assessed the sclerostin concentration in 33 women with PsA comparing the obtained results with a group of 28 women with RA and 35 healthy women of the control group. No statistically significant differences were found between the groups for sclerostin [91]. Fassio et al. assessed the efficacy of treatment with secukinumab (anti-IL-17) in a group of 28 patients, as compared to a control group (n = 43), at a follow-up after 1, 3 and 6 months, and demonstrated that it may have an effect on the activity of osteocytes, including Wnt/β-catenin pathway inhibitors, which may suggest the potential of the drug to inhibit local excessive bone proliferation, being a typical component of PsA. The treatment used did not cause a statistically significant increase in sclerostin concentration in the study groups in comparison to the control group [92]. In 2019, Diani et al. compared a group of 50 patients with psoriasis with a group of 50 patients with PsA and a group of 20 healthy individuals in order to identify potential differences in the concentrations of predictive osteoimmunological biomarkers. The study mentioned above showed that both the patients with psoriasis and the patients with PsA had higher sclerostin concentrations than the individuals from the control group. Moreover, the study provided evidence for a positive correlation between sclerostin concentration and the duration of psoriasis [93]. Tasende et al. conducted a study on a group of 45 patients, including 15 individuals with a diagnosis of PsA, 8 individuals with RA, 15 patients with chronic arthritis and 4 patients diagnosed with AS. In the above-mentioned study, there was no statistically significant increase in mRNA expression in synovium, or in sclerostin concentration in patients with PsA in comparison to patients with RA, chronic arthritis or ankylosing spondylitis [94].

## 6. The Role of Sclerostin in Ankylosing Spondylitis

Ankylosing spondylitis (AS) is an inflammatory disease affecting sacroiliac joints, spinal joints, fibrous rings and spinal ligaments. The underlying inflammatory process results in gradual spinal damage. The aetiology of this condition has not been fully elucidated [15,95,96]. AS is characterised by chronic inflammation and bone remodelling as a result of osteogenesis, mainly in the axial skeleton [97]. The link between inflammation and bone remodelling has not been fully explained. TNF-α as a pro-inflammatory cytokine in AS is responsible for the induction of Dkk-1 and sclerostin [98], which in turn downregulate bone formation via the inhibition of Wnt and bone morphogenic proteins (BMPs) [28]. However, bone formation is also responsible for mechanical load, which stimulates osteocytes to produce BMP, and thus activates the Wnt pathway and inhibits the production of Dkk-1 and sclerostin [99]. In vivo studies in a mouse model of SpA demonstrated that mechanical stress causes enthesitis and bone remodelling [100].

The first publication discussing the role of sclerostin in the course of AS appeared in 2009. Appel et al. reported a lower sclerostin concentration in patients diagnosed with AS in comparison to a control group of healthy volunteers. Additionally, they found that a low sclerostin level was correlated with syndesmophyte formation [101]. In the years to follow, some publications reported decreased [102,103,104,105,106,107,108,109,110,111,112] or increased [113,114,115] sclerostin levels in the course of AS, while according to other reports its values did not differ from those found in the control group [116,117,118]. Consequently, the role of sclerostin remains unclear. A low sclerostin concentration is correlated with an increased Dkk1 concentration, which confirms its inhibitory effect on the Wnt/β-catenin pathway [101,102]. The association of sclerostin and syndesmophyte formation was also confirmed by Heiland et al. [102,119]. However, this correlation remains unclear [103,120]. Korkosz et al. demonstrated that sclerostin levels in patients treated with TNF inhibitor remained unchanged [121]. Similar observations were made by Ustun et al., who found that sclerostin by itself did not induce inflammation or damage which could be visualised via radiological examination [122,123]. In a study assessing sclerostin concentration during 12-week therapy with apremilast, a PDE4 inhibitor, a significant reduction in sclerostin levels was seen. However, the clinical relevance of this report remains uncertain [120]. 

In Table 1, detailed data are presented comparing patients and healthy controls, as well as the type of assay used.

Differences in the results obtained between individual studies may result from the use of tests from different manufacturers, despite the use of the same testing technique in most cases, as well as the accompanying additional diseases. The concentration of sclerostin largely depends on the test used for measurement. Delanaye P. et al. showed that the concentrations obtained using the R&D Systems and MesoScaleDiscovery tests were lower than those obtained using Biomedica or TECO Medical, which means that the results must be interpreted with great caution [124]. Moreover, the results were influenced by the heterogeneity of the study groups, as well as the use of various exclusion criteria from the studies, which are presented in Table S1 of the Supplementary Materials. So far, higher sclerostin concentrations have been observed in older patients and patients with chronic kidney disease or type 2 diabetes mellitus [125,126].

## 7. Conclusions

Among numerous clinical aspects connected with the course of rheumatoid arthritis, disorders of bone metabolism and resultant complications significantly contribute to a worse prognosis as negative regulators of bone growth. It may influence the development of osteoporosis and erosions. In both RA and AS, TNF-α appears to play a key role in sclerostin levels and increased osteoclast activity, but this is only reflected in studies in RA. In AS, it appears that mechanical stress has a greater effect on inhibiting sclerostin formation than TNF-α has on its increased production. It seems that in patients with PsA, the concentration of IL-17 and IL-23 should be of key importance for the concentration of sclerostin and the Wnt/β-catenin pathway itself; however, previous studies comparing groups of patients with PsA with a control group of healthy people do not confirm this, which may also mean that other factors may play a role. For patients with SLE, the sclerostin concentration appears to be a promising biomarker associated with lupus nephropathy or increased cardiovascular risk, which is related to an increased production of IFN-α and effects on T lymphocytes. In spondyloarthropathy, the significance of sclerostin remains unclear and requires further investigation. The Wnt/β-catenin pathway is a key regulator in bone remodelling, but its role requires further research to gain a better understanding of this issue.

## Figures and Tables

**Table 1:** Summary of study results comparing a group of patients to healthy controls.

Authors (Ref)	Number of Patients	Number of Healthy Controls	Patient Group		Age (Mean ± SD)	Level of Sclerostin	Assay Name	Producer	Tissue
			Number of Females	Number of Males					
**RA**									
Dhakad U et al. [62]	47	28	47	0	32.7 ± 6.8	Elevated	ELISA	No data	Serum
El-Bakry S et al. [63]	31	10	28	3	40 (without SD)	Elevated	ELISA	Biomedica	Serum
Singh A et al. [64]	50	50	41	9	41.30 ± 12.971	Elevated	ELISA	RayBio	Serum
Mehaney DA et al. [65]	40	40	33	7	46.7 ± 13.6	No difference	ELISA	TECO Medical	Serum
Świerkot J et al. [66]	27	12	27	0	54.7 (without SD)	No difference	ELISA	TECO Medical	Serum
Seror R et al. [67]	694	453	694	0	48.5 (without SD)	Decreased	ELISA	Biomedica	Serum
**SLE**									
Fayed A et al. [80]	100	50	100	0	25.9 ± 5.8	Elevated	ELISA	Quantikine	Serum
Garcia-de los Ríos C et al. [83]	68	No control group	68	0	43.8 ± 11.0	Elevated	ELISA	BI-20472	Serum
**PsA**									
Fassio A et al. [91]	33	35	33	0	58.8 ± 8.8	No difference	ELISA	Biomedica	Serum
Fassio A et al. [92]	28	43	18	10	57 ± 10	No difference	ELISA	Biomedica	Serum
Diani M et al. [93]	50	20	11	39	48 (without SD)	No difference	ELISA	Quantikine	Serum
Pinto Tasende JA et al. [94]	15	No control group	5	10	48.0 (without SD)	No difference	ELISA Quantitative real-time PCR	DAS Superscript^®^ VILO (Thermo Fisher Scientific, Waltham, MA, USA)	Serum Synovial tissue
**AS**									
Appel H et al. [101]	46	50	16	30	No data	Decreased	ELISA	R&D Systems	Serum
Heiland GR et al. [102]	65	No control group	19	46	35.2 ± 10.4	Decreased	ELISA	No data	Serum
Saad CG et al. [103]	30	36	6	24	35.7 ± 11.0	Decreased	ELISA	Biomedica	Serum
Klingberg E et al. [104]	204	80	87	117	49 (without SD)	Decreased	ELISA	Biomedica	Serum
Sakellariou GT et al. [105]	65	36	4	61	41.3 (without SD)	Decreased	ELISA	Aviscera Bioscience	Serum
Rossini M. et al. [106]	71	70	12	59	Men 43 ± 12 Women 49 ± 12	Decreased	ELISA	Biomedica	Serum
Solmaz D et al. [107]	97	48	21	76	38 ± 14.0	Decreased	ELISA	PeloBiotech, Planegg	Serum
Genre F et al. [108]	119	63	46	73	44.9 ± 11.9	Decreased	ELISA	TECO Medical	Serum
Luchetti MM et al. [109]	45 *	20	20	25	43 (without SD)	Decreased	ELISA ELISA	ICL Lab Inc	Serum Serum
Perrotta FM et al. [110]	40	20	10	30	50 (without SD)	Decreased	ELISA	AUROGENE srl	Serum
Gercik O et al. [111]	55	57	21	34	41 (without SD)	No difference	ELISA	Elabscience	Serum
Iaremenko O et al. [112]	102	15	35	67	38.1 ± 11.2	Decreased	ELISA	Biomedica	Serum
Korkosz M et al. [113]	50—high activity 28—low activity	23	8 No data	42 No data	37.8 ± 11.6 32.0 ± 6.6	Increased No difference	ELISA	Biomedica	Serum
Sun W et al. [115]	88	26	22	66	36.5 ± 13.5	Increased	ELISA	Biomedica	Serum
Sakellariou GT et al. [116]	57	34	4	53	39.1 ± 1.4	No difference	ELISA	Aviscera Bioscience Inc.	Serum
Taylan A et al. [117]	55	33	7	48	36 (without SD)	No difference	ELISA	Biomedica	Serum
Tuylu T et al. [118]	45—syndesmophyte (+) 49—syndesmophyte (−)	68	13 16	32 33	43.9 ± 9.9 40.7 ± 8.7	No difference	ELISA	Biomedica	Serum

* Inflammatory bowel disease (IBD)-associated spondyloarthritis (SpA/IBD).

## Data Availability

No new data were created in this work.

## Supplementary Materials

The following supporting information can be downloaded at: https://www.mdpi.com/article/10.3390/jcm12196248/s1, Table S1: Summary of exclusion criteria.
