# Supplementary material for: The Role of Sclerostin in Rheumatic Diseases: A Review
Source: J Clin Med. 2023 Sep 28;12(19):6248. doi: 10.3390/jcm12196248 (PMC10573925; doi:10.3390/jcm12196248)
Supplement: Supplementary file 1 [file jcm-12-06248-s001.zip › jcm-2592395-supplementary.pdf]

Journal of
Clinical Medicine

MDPI

Authors (Ref)	Exclusion criteria
	**RA**
Dhakad U, et al. [48]	• Other autoimmune diseases,<br>• secondary causes of osteoporosis (such as diabetes, celiac disease, and thyroid disease),<br>• severe Vitamin D deficiency,<br>• chronic liver disease,<br>• chronic kidney disease stage 3 and above,<br>• patients who were on medications that would possibly alter the BMD such as anticonvulsants were excluded from the study,<br>• postmenopausal osteoporosis is a potential confounder.
El-Bakry S, et al. [49]	• Paget disease,<br>• multiple myeloma,<br>• breast cancer,<br>• bone metastasis,<br>• patients who were receiving biological treatment intheform of TNF-a inhibitors during the last 6 months were excluded from this study.
Singh A, et al. [50]	• Age less than 15 years,<br>• patients on treatment of disease modifying anti rheumatic drug,<br>• evidence of steroid use in past 1 month,<br>• calcium supplement in last 3 months,<br>• diabetes mellitus,<br>• chronic kidney disease,<br>• chronic liver disease.
Mehaney DA, et al. [51]	• Diabetes mellitus,<br>• hyperthyroidism,<br>• receiving thyroxin or calcium<br>• replacement therapy or steroid therapy
Świerkot J, et al. [52]	• Pregnancy or breastfeeding,<br>• other systemic diseases of connective tissue besides RA,<br>• clinically significant impairment of hepatic and renal function,<br>• alcohol abuse,<br>• infection with hepatotropic viruses,<br>• infections resistant to therapy,<br>• history of cancer if no cure was achieved,

	• uncontrolled diabetes,<br>• patient unwilling or unable to cooperate.
Seror R, et al. [53]	• Another defined inflammatory rheumatic disease<br>• patients had to be free of any steroids and DMARDs (except within the 15 days before inclusion for DMARDs only).
	**SLE**
Fayed A, et al. [66]	• Any conditions that may cause glomerulopathy including diabetes and essential hypertension,<br>• atients with SLE with a history of lupus nephritis and who showed no evidence of activity.
Garcia-de los Ríos C, et al. [69]	• No data.
	**PsA**
Fassio A, et al. [74]	• Further systemic inflammatory diseases,<br>• active infections,<br>• neoplasms,<br>• metabolic bone diseases,<br>• pregnancy,<br>• current use of biologic therapy,<br>• current use of drugs known to affect bone metabolism,<br>• use of corticosteroids in the last 6 months.
Fassio A, et al. [75]	• Previous treatment with biologic drugs,<br>• other systemic inflammatory diseases,<br>• active infections,<br>• neoplasms,<br>• kidney, liver, endocrine or metabolic bone diseases,<br>• pregnancy,<br>• current use of biologic treatments, corticosteroids or drugs known to affect bone metabolism.
Diani M, et al. [76]	• Pregnancy,<br>• current or previous malignancies,<br>• other acute or chronic inflammatory diseases, infectious diseases (human immunodeficiency virus (HIV), hepatitis B and C, tuberculosis),<br>• other rheumatologic diseases,<br>• primary bone metabolic diseases,<br>• recent bone fractures (within 6 months),

	• anxiety,<br>• psychosis, or depressive disorders,<br>• supplementations, or particular diets (included fasting).
Pinto Tasende JA, et al. [77]	• Macroscopic or microscopic characteristics of the synovial membrane samples presented characteristics different from the underlying pathology (e.g., deposits of urate or PPCD crystals, pigmented villonodular synovitis or vasculitis).
**AS**	
Appel H, et al. [84]	• No data.
Heiland GR, et al. [85]	• None of the patients was treated with tumour necrosis factor (TNF) blockers.
Saad CG, et al. [86]	• Other diseases that may have an effect on bone metabolism such as hyperthyroidism, malabsorption syndrome, kidney and liver failure, alcohol,<br>• prior or current use of bisphosphonates,<br>• current glucocorticoid use (> 5 mg/day) for patients and glucocorticoid use for control group.
Klingberg E, et al. [87]	• Psoriasis,<br>• inflammatory bowel disease,<br>• dementia,<br>• other concomitant rheumatic disease,<br>• difficulties in understanding Swedish.
Sakellariou GT, et al. [88]	• Uncontrolled thyroid disease, primary or secondary hyperparathyroidism and osteoporosis,<br>• severe liver or kidney disease (creatinine clearance <60ml/min/1.73m2),<br>• any malignancy,<br>• alcohol abuse,<br>• use of glucocorticoids in the previous 6 months,<br>• a high in take of NSAIDs according to the recently proposed.
Rossini M. et al. [89]	• Inflammatory bowel disease, clinical or laboratory evidence of hepatic, renal or bone metabolic diseases,<br>• treatment with drugs known to interfere with bone or mineral metabolism, including TNF-a blockers, glucocorticoids, and bisphosphonates.
Solmaz D, et al. [90]	• Uncontrolled hypertension,<br>• severe hepatic and kidney disease (creatinine clearance <60 mL/min),<br>• severe obesity (body mass index >35 kg/m2),<br>• malignancy,

	• patients receiving TNF inhibitor (TNFi).
Genre F, et al. [91]	• Cardiovascular events,<br>• diabetes mellitus,<br>• chronic kidney disease,<br>• IBD,<br>• psoriasis.
Luchetti MM, et al. [92]	• TNF inhibitors upon entry in the study and/or for ≥ 3 months before the collection of biological samples,<br>• use of cyclooxygenase-2 inhibitors, which could induce IBD flare, was not allowed, except occasionally for the relief of pain.
Perrotta FM, et al. [93]	• Age ≤ 18 years,<br>• the presence of history of bone fractures in the previous 24 months,<br>• no treatment with bisphosphonate agents.
Gercik O, et al. [94]	• Age ≤ 18 years,<br>• pregnant or lactating,<br>• end-stage renal disease,<br>• malignant tumor,<br>• cirrhosis,<br>• hyperthyroidism,<br>• hyperparathyroidism or Cushing's syndrome,<br>• using tumor necrosis factor inhibitors (TNFi).
Iaremenko O, et al. [95]	• A history of the other rheumatic diseases,<br>• spinal surgery,<br>• spinal tumor.
Korkosz M, et al. [96]	• No data.
Sun W, et al. [98]	• Low phosphorus rickets and osteoporosis with pathological fracture as well as malignant diseases.
Sakellariou GT, et al. [99]	• History of osteoporosis,<br>• severe liver or kidney dysfunction,<br>• history of cardiovascular disease, diabetes mellitus or metabolic syndrome,<br>• any malignancy,<br>• previous use of biphosphonates or other anti-osteoporotic treatment,<br>• use of glucocorticoids in the previous 6 months,

	• a high intake of non-steroidal anti-inflammatory drugs (NSAIDs) according to the recently proposed.
Taylan A, et al. [100]	• History of neuroendocrine disorder (thyroid, parathyroid disorder, and anticonvulsant usage),<br>• chronic renal and liver disease,<br>• systemic steroid usage, menopause,<br>• cigarette smoking,<br>• excessive alcohol usage.
Tuylu T, et al. [101]	• Renal impairment (serum creatinine.1.4 mg/dl),<br>• patients who were treated with glucocorticoids during the previous four weeks.

Supplementary materials – Table 1. Summary of exclusion criteria.
